# Erratum to: Shenmai injection as an adjuvant treatment for chronic cor pulmonale heart failure: a systematic review and meta-analysis of randomized controlled trials

**DOI:** 10.1186/s12906-015-0953-4

**Published:** 2015-12-03

**Authors:** Liwei Shi, Yanming Xie, Xing Liao, Yan Chai, Yanhua Luo

**Affiliations:** Institute of Basic Research in Clinical Medicine, China Academy of Chinese Medical Sciences, Beijing, China; Guang’an men Hospital, China Academy of Chinese Medical Sciences, Beijing, China; Department of Epidemiology, University of California-Los Angeles, Los Angeles, CA USA

After publication of the original article [1] it was brought to our attention that Additional files 1 and 3 contained the wrong citations. The correct files have been attached to this Erratum.

## Additional files

Additional file 1:
**PRISMA 2009 checklist for this systematic review and meta-analysis.** (DOC 191 kb) Additional file 3: Table S1. Characteristics of 27 included studies on SM for chronic cor pulmonale heart failure. (DOC 75 kb)
