# Supplementary material for: Shenmai injection as an adjuvant treatment for chronic cor pulmonale heart failure: a systematic review and meta-analysis of randomized controlled trials
Source: BMC Complement Altern Med. 2015 Nov 24;15:418. doi: 10.1186/s12906-015-0939-2 (PMC4659214; doi:10.1186/s12906-015-0939-2)
Supplement: Additional file 1: — PRISMA 2009 checklist for this systematic review and meta-analysis. (DOC 187 kb) [file 12906_2015_939_MOESM1_ESM.doc]

| **Section/topic** | **#** | **Checklist item** | **Reported on page #** |
| --- | --- | --- | --- |
| **TITLE** | | |  |
| Title | 1 | Shenmai injection as an adjuvant treatment for chronic cor pulmonale heart failure: a systematic review and meta-analysis of randomized controlled trials | 1 |
| **ABSTRACT** | | |  |
| Structured summary | 2 | Background: Shenmai injection (SM), as a traditional Chinese medicine injection, is widely used for chronic cor pulmonale heart failure in mainland China. It is essential to systematically assess the efficacy and safety of SM as an adjuvant treatment for chronic cor pulmonale heart failure. **Methods:** Eight English and Chinese electronic databases were searched, from inception to December 2014, to identify randomized controlled trials (RCTs) of SM for chronic cor pulmonale heart failure. The Cochrane Risk of Bias tool was used to evaluate the methodological quality of eligible studies. Meta-analysis was performed by Review Manager 5.2.  **Results:** Twenty-seven RCTs with 2045 participants were identified. The methodological quality of the included studies was generally low. Only one trial reported data on death. None of the included trials reported quality of life. The meta-analysis indicated that compared to conventional treatment, the combination of SM and conventional treatment was more effective in terms of the New York Heart Association classification (RR, 1.26; 95% CI, 1.20-1.32; P<0.00001), Left Ventricular Ejection Fraction (MD, 11.33; 95% CI, 8.59-14.07; p<0.00001), partial pressure of oxygen (MD, 1.00; 95% CI, 0.64-1.36; P<0.00001) and partial pressure of carbon dioxide (MD, 0.83; 95%CI, 0.58-1.08; p<0.00001). In addition, two trials reported that SM plus conventional treatment was superior to the conventional treatment alone to reduce B-type natriuretic peptide. No serious adverse drug events or reactions were reported. Conclusions: SM plus conventional treatment appeared to be effective and relatively safe for chronic cor pulmonale heart failure. However, due to the generally low methodological quality and small sample size, this review didn’t find evidence to support routine use of SM as an adjuvant treatment for chronic cor pulmonale heart failure.Keywords: Shenmai injection (SM), Chronic cor pulmonale heart failure, Systematic review, Meta-analysis | 2-3 |
| **INTRODUCTION** | | |  |
| Rationale | 3 | Chronic cor pulmonale, a common type of heart disease, is a rising major public health problem around the world. The treatment for chronic cor pulmonale is still unsatisfactory now. Shenmai injection (SM), as a traditional Chinese medicine injection, derives from a traditional decoction named Shenmai yin prescribed by a famous traditional Chinese medicine doctor named Si-miao Sun in the Tang Dynasty. It is a traditional Chinese herbal medicine that has been used for patients with qi-yin deficiency in China for about 1500 years, consisting of Panax ginseng and Ophiopogon japonicus. Shenmai injection (SM) has been approved by China Food and Drug Administration (CFDA) on the market for chronic cor pulmonale heart failure since 1995. It is widely used for chronic cor pulmonale heart failure with qi-yin deficiency. A large number of clinical trials showed that SM benefited patients with chronic cor pulmonale heart failure. The previous systematic review reported that SM might have potential therapeutic effects for chronic pulmonary heart disease. However, evidence was very limited on the efficacy and safety of SM for chronic cor pulmonale heart failure of different severity, due to lack of detailed description about the New York Heart Association (NYHA) classification and poor methodological quality. | 3-4 |
| Objectives | 4 | To assess systematically and critically the efficacy and safety of SM as an adjuvant treatment for chronic cor pulmonale heart failure. | 4 |
| **METHODS** | | |  |
| Protocol and registration | 5 | International clinical trial registry by U.S. national institutes of health (http://clinical trials.gov/). | 6 |
| Eligibility criteria | 6 | Inclusion criteria: The trials that met the following inclusion criteria were included: (1) types of studies: all randomized controlled trials (RCTs) of SM for chronic cor pulmonale heart failure; (2) types of participants: patients who were diagnosed as chronic cor pulmonale heart failure with NYHA classification from I to IV; (3) types of interventions: SM was combined with conventional medical treatment, compared to conventional medical treatment alone, and SM was used by intravenous drip; (4) the primary outcome measures included death from any cause during the scheduled treatment and follow-up and quality of life as measured by various instruments; the secondary outcome measures included NYHA classification, LVEF, BNP, partial pressure of oxygen (PaO2) and partial pressure of carbon dioxide (PaCO2), and adverse drug events (ADEs) or adverse drug reactions (ADRs) during the scheduled treatment and follow-up; (5) the studies contained available and relevant data for meta-analysis; (6) the studies were available in any language. Any disagreement was resolved by consensus or discussion with a third party (YM Xie and X Liao).Exclusion criteria: If involved any condition of the followings, trials were excluded: (1) duplicated publications; (2) data was unavailable or incorrect, or no relevant data for meta-analysis; (3) quasi-randomized controlled clinical trials (that is, allocation using alternation, the sequence of admission, case record numbers, dates of birth), non-randomized controlled clinical trials; (4) the patients were diagnosed as chronic cor pulmonale heart failure with unclear NYHA classification; (5) patients with comorbidities of coronary heart disease or severe liver and kidney diseases; (6) combined with any other herbal medicines in experimental or control group during the treatment. | 5-6 |
| Information sources | 7 | A comprehensive search was performed to identify all published randomized controlled clinical trials. All relevant studies were sought regardless of any language. The relevant trials were retrieved from the following databases: the Cochrane Central Register of Controlled Trials (CENTRAL) on the Cochrane Library (Issue 10 of 12, December 2014); PubMed (1966 to December 2014); EMBASE (1980 to December 2014); Chinese Biomedical Literature Database (CBM, 1978 to October 2014); Chinese Scientific Journal Database (VIP, 1989 to October 2014); Wan Fang Database (1990 to October 2014); and Chinese National Knowledge Infrastructure (CNKI, 1979 to October 2014). Ongoing registered clinical trials were searched in the Clinical Trials, gov (http: // clinical trials. gov/). All of these searches ended on 7 December, 2014. The following search terms were used individually or combined: “chronic cor pulmonale heart failure”, “chronic cor pulmonale with cardiac dysfunction”, “chronic cor pulmonale with cardiac insufficiency”, “Shenmai injection”, and “Shenmai”. The literature search was performed independently by two authors (LW Shi and YH Luo), and disagreements were resolved by discussion. The details for the full search strategy were listed in a flow diagram. | 6 |
| Search | 8 | **Four English electronic databases:** Cochrane Central Register of Controlled Trials (CENTRAL) on the Cochrane Library, PubMed, EMBASE, the Clinical Trials, gov.  **Four Chinese electronic databases:** Chinese Biomedical Literature Database (CBM), Chinese Scientific Journal Database (VIP), Wan Fang Database, Chinese National Knowledge Infrastructure (CNKI). | 6  (Additional file 2) |
| Study selection | 9 | Two authors (LW Shi and YH Luo) independently conducted the literature searching and study selection. | 5-6 |
| Data collection process | 10 | Two authors (LW Shi and YH Luo) independently extracted information on patients, methods, interventions, outcomes and results using a data extraction form designed for this review. For dichotomous outcomes, we extracted the number of events and the total number of participants for each group. For continuous outcomes, we abstracted mean changes, standard deviations and the total number for each study. The data extraction form included the following items: (1) general information: title, authors, year of publication, and source; (2) trial characteristics: design, duration of follow up, method of randomization, allocation concealment, incomplete outcome data, blinding (patients, people administering treatment, outcome assessors); (3) intervention(s): intervention(s) (dose, usage, duration and frequency), comparison intervention(s) (dose, usage, duration and frequency); (4) patients: total number and number in both groups, baseline characteristics, diagnostic criteria, withdrawals and losses to follow up (reasons, description); (5) outcomes: outcomes specified above, any other outcomes assessed, length of follow up, quality of reporting of outcomes. Any disagreement was settled by discussion or by consulting a third author (YM Xie and X Liao). | 6-7 |
| Data items | 11 | **The primary outcome measures:** Death from any cause during the scheduled treatment and follow-up and quality of life as measured by various instruments.  **The secondary outcome measures:** New York Heart Association (NYHA) classification, Left Ventricular Ejection Fraction (LVEF), B-type natriuretic peptide (BNP), partial pressure of oxygen (PaO2) and partial pressure of carbon dioxide (PaCO2), and adverse drug events (ADEs) or adverse drug reactions (ADRs) during the scheduled treatment and follow-up. | 5 |
| Risk of bias in individual studies | 12 | The methodological quality of trials was assessed by Review Manager Version 5.2 from the Cochrane Handbook for Systematic Review of Interventions. We assessed the methodological quality of each trial in terms of random sequence generation (selection bias), allocation concealment (selection bias), blinding of participants and personnel (performance bias), blinding of outcome assessment (detection bias), incomplete outcome date (attrition bias), selective reporting (reporting bias) and other bias. Then we classified each quality component as “low”, “high”, “unclear”. If all the items were in low risk of bias, the trial would be categorized into low risk of bias, if one or more items were in high or unclear risk of bias, the trial would be categorized into high or unclear risk of bias, respectively. Low risk of bias represented a good quality. Any disagreement was settled by discussion or by consulting a third author (YM Xie and X Liao). | 7 |
| Summary measures | 13 | Meta-analysis was performed by RevMan 5.2. For measurement outcomes, D-value of the pre and post treatment was used for statistical analysis. Dichotomous outcomes were presented as risk ratio (RR) and 95% confidence intervals (CI), while continuous outcomes were expressed as mean difference (MD) and 95% confidence intervals (CI). | 7 |
| Synthesis of results | 14 | The I-square (I^2^) statistic, a quantitative measure of inconsistency across studies, was used to assess heterogeneity. If the I^2^ statistic was equal to or less than 50%, it suggested that there was minor heterogeneity, fixed effect model would be used to perform a meta-analysis. If the I^2^ statistic was between 50% to 90%, it suggested that there was substantial heterogeneity, random effect model would be used to perform a meta-analysis. If the I^2^ statistic was equal to or greater than 90%, it suggested that there was considerable heterogeneity, the meta-analysis wouldn’t be performed, and instead the results of the trials would be described. Subgroup analyses were performed to evaluate the effects of SM plus conventional treatment in improving NYHA classification, LVEF or PaO2, and decreasing BNP or PaCO2. If death and quality of life were reported in the included trials, subgroup analyses were conducted to assess the effects of SM plus conventional treatment on death and quality of life. | 7-8 |

Page 1 of 2

| **Section/topic** | **#** | **Checklist item** | **Reported on page #** |
| --- | --- | --- | --- |
| Risk of bias across studies | 15 | Publication bias was assessed using a funnel plot if the group included more than 10 trials. | 8 |
| Additional analyses | 16 | Sensitivity analysis was performed to examine the effects of excluding study subgroups, such as those studies with lower methodological quality. | 8 |
| **RESULTS** | | |  |
| Study selection | 17 | A total of 1434 records were identified after removing duplicates. During the preliminary screening of the titles and abstracts, 1373 records were removed. After full-texts screening, 34 trials were excluded with the following reasons: participants didn’t meet the inclusion criteria (n=17), duplication (n=3), no control group (n=5), quasi-RCT (n=3), non-RCT (n=1), no data available for extraction (n = 3), and without reporting relevant outcomes (n=2). Finally, 27 RCTs of SM for chronic cor pulmonale heart failure were included in this systematic review. The detailed process of search and selection was shown in [figure 1](#Fig1). | 8  (Figure 1) |
| Study characteristics | 18 | A total of 27 RCTs with 2045 participants were included in this review. The treatment group consisted of 1055 patients, while the control group consisted of 990 patients. All the included trials were conducted in China, and published in Chinese. Sample size of the included trials ranged from 44 to 120, with the average number of 76 per trial. Only four trials [14,19,32,34] included more than 100 patients. About 65% of the participants were males. Although there was a wide variation in the age of subjects (34-89 years), the included patients were mainly elderly. Fifteen trials [14-17,21-25,27-29,32,38,39] reported the course of chronic cor pulmonale with a wide variation. All patients were diagnosed as chronic cor pulmonale heart failure with NYHA classification from I to IV, most of who were diagnosed as chronic cor pulmonale heart failure with NYHA classification from II to IV.  The treatment group used SM combined with the same conventional treatment as control group. The dose of SM varied from 10ml to 100ml, most of which was from 30ml to 60ml. Shenmai injection (SM) was administered as intravenous drip in all included trials. Two trials [34,36] did not mention solvent. One trial [14] used SM 1.0ml/(kg·d) by intravenous drip without solvent. One trial [15] used SM 100ml by intravenous drip without solvent. The other 23 trials used 0.9% normal saline or 5% glucose with the volume from 100ml to 250ml as the solvent for SM. Shenmai injection (SM) was used once daily in all included trials, except one trial [32], in which SM was used twice daily. The intervention time ranged from seven days to fifteen days. The control group used conventional medical treatment alone, including antibiotics, expectorants, antiasthmatic drugs, oxygen therapy, diuretics, digitalis, vasodilators, antiarrhythic drugs and anticoagulants.  Only one trial [16] reported death. None of the included trials reported quality of life. Twenty-five trials [15-39] of the 27 included trials reported NYHA classification. Five trials [13,14,21,23,35] reported LVEF. Four trials [29,32,36,38] reported PaO2 and PaCO2. Only two trials [13,14] used BNP as outcome measure. Six [16,18,29,32,35,37] out of the 27 included trials reported a total of 13 patients with ADEs or ADRs, including pain, dizziness, palpitation, rash, dry mouth, poor appetite, headache, nausea and vomiting. The details of study characteristics were shown in additional file 1. | 8-9  (Additional file 3) |
| Risk of bias within studies | 19 | The methodological quality of the included trials was generally poor. Two trials [14,35] reported that random sequence was generated by a random number table, the remaining 25 trials only mentioned random allocation without any description about the method of randomization. None of the trials described the allocation concealment. Blinding of participants and personnel and blinding of outcome assessment were not mentioned in all included trials. None of the included trials reported withdrawals or dropouts or performed an intention-to-treat analysis. Selective reporting was generally unclear due to the inaccessibility to the trial protocol. Other potential sources of bias were unclear. None of the trials had a pre-trial estimation of sample size. Therefore, the overall quality rating of all the included trials was graded as high risk of bias. More details of the trials were presented in Figure 2. | 9-10  (Figure 2) |
| Results of individual studies | 20 | **Primary outcome measures**  **Death**  Only one trial [16] reported death. This trial reported that five patients were in a critical condition and eventually died during hospitalization, two from treatment group and three from control group. However, there was no statistically significant difference between SM plus conventional treatment and conventional treatment alone on death (RR, 0.65; 95% CI, 0.11-3.67; P=0.62). Other trials didn’t report death during the scheduled treatment. None of the trials reported the continued follow up after the treatment period.  **Quality of life**  None of the included trials performed the assessment of quality of life.  **Secondary outcome measures**  **New York Heart Association (NYHA) classification**  A total of 25 trials [15-39] with 1839 patients investigated the effect of SM plus conventional treatment in improving NYHA classification in patients with chronic cor pulmonale heart failure. NYHA classification as a dichotomous outcome, the number of responders and the total number of participants for each group were extracted to analyze risk ratio (RR), which was calculated as the ratio between the proportion of responders in treatment group and the proportion of responders in control group. Responders were defined as an improvement of at least one class on NYHA classification. The I-square (I^2^) statistic based on the data for NYHA classification showed that there was no significant heterogeneity among 25 trials (I^2^=0%, P=0.89), and fixed effect model was used to pool the results of these trials. The pooled analysis of 25 trials indicated that SM plus conventional treatment showed greater improvement on NYHA classification than conventional treatment alone (RR, 1.26; 95% CI, 1.20-1.32; P<0.00001, Figure 4).  **Left Ventricular Ejection Fraction (LVEF)**  A total of five trials with 408 patients [13,14,21,23,35] assessed the effect of SM plus conventional treatment in improving LVEF in patients with chronic cor pulmonale heart failure. The I-square (I^2^) statistic based on the data for LVEF showed that there was substantial heterogeneity among these trials (I^2^=68%, P=0.01), and random effect model was used to pool the results of these trials. The meta-analysis indicated that there was a statistically significant difference between SM plus conventional treatment and conventional treatment alone on LVEF (MD, 11.33; 95% CI, 8.59-14.07; p<0.00001, Figure 5).  **B-type natriuretic peptide (BNP)**  Only two trials with 206 patients [13,14] assessed the effect of SM plus conventional treatment in decreasing BNP in patients with chronic cor pulmonale heart failure. The I-square (I^2^) statistic based on the data for BNP showed that there was considerable heterogeneity between two trials (I^2^=94%, P<0.0001). One trial [14] reported that SM plus conventional treatment had better effect than conventional treatment alone on BNP (MD, 88.70; 95% CI, 74.28-103.12; p<0.00001), the other trial [13] reported that SM plus conventional treatment was superior to conventional treatment alone to reduce BNP (MD, 165; 95% CI, 130.93-199.07; p<0.00001)  **Partial pressure of oxygen (PaO2) and carbon dioxide (PaCO2)**  Four trials with 301 patients [29,32,36,38] assessed the effects of SM plus conventional treatment in increasing PaO2 and decreasing PaCO2 in patients with chronic cor pulmonale heart failure. The I-square (I^2^) statistic based on the data for PaO2 showed that there was substantial heterogeneity among these trials (I^2^=84%, p=0.0002), while the I-square (I^2^) statistic based on the data for PaCO2 showed that there was no significant heterogeneity among these trials (I^2^=48%, p=0.13). Random effect model was used to pool the results of these trials. The pooled analysis indicated that there was a statistically significant difference between SM plus conventional treatment and conventional treatment alone on PaO2 (MD, 1.00; 95% CI, 0.64-1.36; P<0.00001, Figure 6) and PaCO2 (MD, 0.83; 95%CI, 0.58-1.08; p<0.00001, Figure 6). It seemed that SM as an adjunct to conventional medication could improve respiratory function.  **Adverse drug events or reactions (ADEs or ADRs)**  Six trials [16,18,29,32,35,37] reported 13 patients with ADEs or ADRs, eleven patients in treatment group and two patients in control group. One trial [16] reported that in treatment group three patients suffered pain at the injection site and two patients suffered dizziness and palpitation, who recovered after slowing the infusion speed of SM. There was one trial [18] reporting one patient suffered dry mouth, who recovered after slowing the infusion speed, which we regarded as ADE due to unclear causal relationship judgment between the event and SM. One trial [29] reported that ADR of rash appeared in one patient in treatment group, which disappeared after withdrawal of SM. One trial [32] reported that ADR of scattered red rash occurred in one patient in treatment group, which disappeared after withdrawal of SM. One trial [35] reported that two patients in treatment group suffered poor appetite, who recovered without special treatment. One trial [37] reported that one patient in treatment group suffered palpitation, and two patients in control group suffered mild headache, palpitation, nausea and vomiting, and poor appetite. After slowing the infusion speed, the patients recovered. We regarded these as ADEs, as this trial [37] didn’t report any other details. Eleven trials clearly reported that no ADEs or ADRs occurred in their trials [15,17,19-21,23,25,26,31,34,38]. The remaining ten trials provided no data regarding ADEs or ADRs [13,14,22,24,27,28,30,33,36,39]. | 10-13  (Figures 4-6 ) |
| Synthesis of results | 21 | **Death and Quality of life:** Only one trial [16] reported death. However, there was no statistically significant difference between SM plus conventional treatment and conventional treatment alone on death (RR, 0.65; 95% CI, 0.11-3.67; P=0.62). None of the included trials performed the assessment of quality of life.  **New York Heart Association (NYHA) classification:** A total of 25 trials [15-39] with 1839 patients investigated the effect of SM plus conventional treatment in improving NYHA classification in patients with chronic cor pulmonale heart failure. There was no significant heterogeneity among 25 trials (I^2^=0%, P=0.89), and fixed effect model was used to pool the results of these trials. The pooled analysis of 25 trials indicated that SM plus conventional treatment showed greater improvement on NYHA classification than conventional treatment alone (RR, 1.26; 95% CI, 1.20-1.32; P<0.00001, Figure 4).  **Left Ventricular Ejection Fraction(LVEF):** A total of five trials with 408 patients [13,14,21,23,35] assessed the effect of SM plus conventional treatment in improving LVEF in patients with chronic cor pulmonale heart failure. There was substantial heterogeneity among these trials (I^2^=68%, P=0.01), and random effect model was used to pool the results of these trials. The meta-analysis indicated that there was a statistically significant difference between SM plus conventional treatment and conventional treatment alone on LVEF (MD, 11.33; 95% CI, 8.59-14.07; p<0.00001, Figure 5).  **B-type natriuretic peptide (BNP):** Only two trials with 206 patients [13,14] assessed the effect of SM plus conventional treatment in decreasing BNP in patients with chronic cor pulmonale heart failure. There was considerable heterogeneity between two trials (I^2^=94%, P<0.0001). One trial [14] reported that SM plus conventional treatment had better effect than conventional treatment alone on BNP (MD, 88.70; 95% CI, 74.28-103.12; p<0.00001), the other trial [13] reported that SM plus conventional treatment was superior to conventional treatment alone to reduce BNP (MD, 165; 95% CI, 130.93-199.07; p<0.00001) .  **Partial pressure of oxygen (PaO2) and carbon dioxide(PaCO2):** Four trials with 301 patients [29,32,36,38] assessed the effects of SM plus conventional treatment in increasing PaO2 and decreasing PaCO2 in patients with chronic cor pulmonale heart failure. The I-square (I^2^) statistic based on the data for PaO2 showed that there was substantial heterogeneity among these trials (I^2^=84%, p=0.0002), while the I-square (I^2^) statistic based on the data for PaCO2 showed that there was no significant heterogeneity among these trials (I^2^=48%, p=0.13). Random effect model was used to pool the results of these trials. The pooled analysis indicated that there was a statistically significant difference between SM plus conventional treatment and conventional treatment alone on PaO2 (MD, 1.00; 95% CI, 0.64-1.36; P<0.00001, Figure 6) and PaCO2 (MD, 0.83; 95%CI, 0.58-1.08; p<0.00001, Figure 6). It seemed that SM as an adjunct to conventional medication could improve respiratory function.  **Adverse drug events or reactions (ADEs or ADRs):** Six trials [16,18,29,32,35,37] reported 13 patients with ADEs or ADRs, eleven patients in treatment group and two patients in control group. Eleven trials clearly reported that no ADEs or ADRs occurred in their trials [15,17,19-21,23,25,26,31,34,38]. The remaining ten trials provided no data regarding ADEs or ADRs [13,14,22,24,27,28,30,33,36,39]. | 10-13  (Figures  4-6 ) |
| Risk of bias across studies | 22 | Publication bias was assessed using a funnel plot based on the NYHA classification reported in 25 trials [15-39]. The funnel plot was asymmetrical indicating that potential publication bias might influence the results of this review. The publication bias might result from the following reasons: small sample size, poor quality and a high proportion of positive results. Funnel plot based on the data for the NYHA classification was elaborated in Figure 3. | 10  (Figure 3) |
| Additional analysis | 23 | Sensitivity analysis was not performed due to all included trials with generally low methodological quality. | 10 |
| **DISCUSSION** | | |  |
| Summary of evidence | 24 | This is the first comprehensive systematic review and meta-analysis to assess the effects of SM as an adjuvant treatment for chronic cor pulmonale heart failure with NYHA classification from I to IV. In this systematic review, only one trial reported death, and none of the included trials reported quality of life. Thus evidence was limited to make a conclusion on death and quality of life. Due to a limited number of trials, poor methodological quality and significant heterogeneity among these trials, it was hard to assess the effects of SM as an adjuvant treatment in improving LVEF, PaO2 and decreasing BNP, PaCO2. The main finding of present review was that SM combined with conventional treatment appeared to be more effective in improving NYHA classification than conventional treatment alone. However, due to generally poor methodological quality, small sample size and publication bias, there was no evidence to support the routine use of SM as an adjuvant treatment for chronic cor pulmonale heart failure. Another finding indicated that SM seemed generally safe, but evidence was limited to make a conclusion on the issue of safety because only 63% studies mentioned the ADEs or ADRs. | 13 |
| Limitations | 25 | **Limitations**  Although the meta-analysis suggested that SM could have potential therapeutic effects and be relatively safe for chronic cor pulmonale heart failure, a number of inherent and methodological weaknesses should be addressed.  Firstly, randomization is necessary to avoid selection bias. However, only two trials [14,35] of the included trials provided specific information on how the random allocation was generated. None of the included trials reported the allocation concealment. Indeed, inadequate allocation concealment results in exaggerated estimates of treatment effect. We didn't contact the authors for the method of randomization due to limited condition. Therefore, we could not confirm that allocation was truly random and well concealed. None of the trials mentioned blinding. Placebo controlled or no treatment is impossible in clinical trials due to ethic issues, as chronic cor pulmonale heart failure is relatively severe. All the included trials were generally of small sample size, and none of the trials reported the method of calculation of the sample size and mentioned the possibility of a type-II error occurring, which was likely to make results lack of power.  Secondly, none of the trials mentioned ethical issues or whether the participants gave informed consent, except one study [14]. Therefore, it seemed that reports of the trials didn’t conform to the recommendations of the Consolidated Standards of Reporting Trials (CONSORT) statement [40].  Thirdly, the scheduled treatment period ranged from seven to fifteen days in the included trials. All of the trials assessed the efficacy immediately after the termination of the treatment period. None of the trials reported the continued follow up after the treatment period, and tried to investigate the effects that SM improved the prognosis of chronic cor pulmonale heart failure and reduced the times for admission. Therefore, the long-term effect of SM treatment couldn’t be assessed due to lack of long-term follow up.  Fourthly, the outcome measures of all trials were so simple that there was no more or key information for analysis. There was very limited evidence on the primary outcome measures. Only one trial [16] reported death, and none of the trials reported quality of life. The NYHA classification was the most commonly used secondary outcome measure in the included trials, but it was subjective for researchers to describe the improvement of cardiac function class. The other secondary outcome measures, including LVEF, BNP, PaO2 and PaCO2, were reported in a limited number of trials with poor methodological quality and significant heterogeneity. Thus it was hard to assess the effects of SM in improving LVEF, respiratory function and decreasing BNP.  Fifthly, another limitation was publication bias which was assessed by visual inspection of funnel plot. The funnel plot was asymmetrical suggesting the possibility of publication bias. Some researchers [41] reported that some Asian countries including China published unusually high proportions of positive results. In this systematic review, there seemed to have great potential publication bias of SM plus conventional treatment versus conventional treatment on NYHA classification, which might influence the results of this review.  Lastly, special attention should be paid to adverse drug events or reactions. Safety is a fundamental principle in the provision of herbal medicines and herbal products for health care. As more and more adverse drug events of herbal medicines were found and reported. World Health Organization (WHO) published WHO guidelines on safety monitoring of herbal medicines in pharmacovigilance systems in 2004. However, in this systematic review, ten trials [13,14,22,24,27,28,30,33,36,39] did not report the adverse drug events or reactions. Thus, all adverse drug events must be reported by the researchers participating in a clinical trial of SM according to the recommendations of the CONSORT statement [40] in the future.  **Implication for practice**  This systematic review provides weak evidence for the efficacy and safety of SM as an adjuvant treatment for chronic cor pulmonale heart failure, and a clinical recommendation cannot be warranted because of the generally low methodological quality and small sample size of the included studies. Shenmai injection (SM) may have beneficial effects on NYHA classification for chronic cor pulmonale heart failure patients with NYHA classification from I to IV. However, due to generally poor methodological quality, small sample size and publication bias, there is no evidence supporting routine use of SM as an adjunct to conventional medication for chronic cor pulmonale heart failure. Therefore, high-quality RCTs of SM for chronic cor pulmonale heart failure are required to confirm the effects reported in the current systematic review.  Pattern differentiation is a unique TCM concept that summarizes and differentiates the nature, location, and pattern of diseases, which is the essential guide for TCM therapy. The precisely tailoring Chinese herbal prescription for individuals based on each individual pattern can maximize its efficacy. For example, one high-quality RCT of TCM as an adjuvant treatment for chronic heart failure indicated that TCM staging-differentiation treatment depending on pattern differentiation as an adjunct to conventional medication showed better effects than western medicine therapy alone [42]. Therefore, we should combine pattern differentiation with western medical diagnosis in modern TCM research, which is beneficial to improve the effectiveness of the interventions [43]. In this systematic review, none of the included trials mentioned the pattern differentiation, except one trial [14], which reported that the target population was diagnosed as chronic cor pulmonale heart failure with the syndrome of qi deficiency and blood stasis. However, Shenmai injection (SM) is mainly used for chronic cor pulmonale heart failure patients with qi-yin deficiency. Therefore, future clinical trials should include patients who were diagnosed as chronic cor pulmonale heart failure with qi-yin deficiency.  **Implication for future research**  Clinical trials with both high methodological quality and large sample size are required to assess the efficacy and safety of SM as one adjuvant treatment for chronic cor pulmonale heart failure. Sample size should be calculated by the proper statistical method and power or type-II errors should be assessed. Further RCTs of SM for chronic cor pulmonale heart failure should consider more clinically relevant and objective outcome measures, such as death. Researchers of TCM should pay greater attention to the methodological issues including randomization, allocation concealment and blinding. The quality of reporting of future trials should be improved and reports of the trials should conform to the recommendations of the CONSORT statement [40]. For better evaluating the safety of traditional Chinese medicine injection, it is essential to establish a clear monitoring and reporting system for the adverse effects of traditional Chinese medicine injection. Future trials should also give consideration to including long-term evaluation of effectiveness and adverse effects of SM. | 14-17 |
| Conclusions | 26 | Shenmai injection (SM) combined with conventional treatment appeared to be effective and relatively safe for chronic cor pulmonale heart failure with NYHA classification from I to IV. However, currently there was no evidence supporting routine use of SM as an adjunct to conventional medication for chronic cor pulmonale heart failure due to the generally low quality and small sample size of the included trials. Therefore, the efficacy and safety of SM as an adjuvant treatment for chronic cor pulmonale heart failure remain to be determined by methodologically rigorous trials. | 17 |
| **FUNDING** | | |  |
| Funding | 27 | The study was financially supported by the Ninth-Science Foundation of Institute of Basic Research in Clinical Medicine, China Academy of Chinese Medical Sciences (No. Z0406); Scientific Research Innovation Team Project of China Academy of Chinese Medical Sciences (No. PY1303 ); National Natural Science Foundation of China (General Program, No. 81202776) and China Postdoctoral Science Foundation project (No.2014T70202 ). | 18 |

*From:*  Moher D, Liberati A, Tetzlaff J, Altman DG, The PRISMA Group (2009). Preferred Reporting Items for Systematic Reviews and Meta-Analyses: The PRISMA Statement. PLoS Med 6(6): e1000097. doi:10.1371/journal.pmed1000097

For more information, visit: **www.prisma-statement.org**.

Page 2 of 2
