# Supplementary material for: Shenmai injection as an adjuvant treatment for chronic cor pulmonale heart failure: a systematic review and meta-analysis of randomized controlled trials
Source: BMC Complement Altern Med. 2015 Nov 24;15:418. doi: 10.1186/s12906-015-0939-2 (PMC4659214; doi:10.1186/s12906-015-0939-2)
Supplement: Additional file 2: — A supplementary file regarding the full search strategies. (DOC 26 kb) [file 12906_2015_939_MOESM2_ESM.doc]

# Full search strategies

Eight English and Chinese electronic databases were searched: the Cochrane Central Register of Controlled Trials (CENTRAL) on the Cochrane Library (Issue 10 of 12, December 2014); PubMed (1966 to December 2014); EMBASE (1980 to December 2014); Chinese Biomedical Literature Database (CBM, 1978 to October 2014); Chinese Scientific Journal Database (VIP, 1989 to October 2014); Wan Fang Database (1990 to October 2014); Chinese National Knowledge Infrastructure (CNKI, 1979 to October 2014); and the Clinical Trials, gov (http: // clinical trials. gov/). All of these searches ended on 7 December, 2014. Search terms for each Chinese electronic database were showed as follows. A total of 1779 records were searched in all Chinese electronic databases. After removing duplicates, 1007 records were identified. During the preliminary screening of the titles and abstracts, 61 records were identified. After full-texts screening, 27 RCTs of SM as an adjuvant treatment for chronic cor pulmonale heart failure were included. In the process of searching, we used more searching words like “fei yuan xing xin zang bing” or “fei xin bing” in the titles and abstracts. Search terms for each English electronic database were showed as follows. The studies involving “Shenmai injection” or “Shenmai” were all identified. A total of 427 records were searched in all English electronic databases. After removing duplicates, 427 records were identified. During the preliminary screening of the titles and abstracts, 427 records were excluded. Eventually, the current systematic review included 27 RCTs of SM as an adjuvant treatment for chronic cor pulmonale heart failure.

Search terms for Chinese electronic databases:

#1  fei yuan xing xin zang bing xin shuai

#2  fei yuan xing xin zang bing xin li shuai jie

#3  fei yuan xing xin zang bing xin gong neng bu quan

#4  fei yuan xing xin zang bing xin gong neng shuai jie

#5  fei xin bing xin shuai

#6  fei xin bing xin li shuai jie

#7  fei xin bing xin gong neng bu quan

#8  fei xin bing xin gong neng shuai jie

#9 #1 OR #2 OR #3 OR #4 OR #5 OR #6 OR #7 OR #8

#10 Shenmai injection

#11 #9 AND #10

Search terms for English electronic databases:

#1 Shenmai

#2 Shenmai injection

#3 #1 OR #2
