# Supplementary material for: Shenmai injection as an adjuvant treatment for chronic cor pulmonale heart failure: a systematic review and meta-analysis of randomized controlled trials
Source: BMC Complement Altern Med. 2015 Nov 24;15:418. doi: 10.1186/s12906-015-0939-2 (PMC4659214; doi:10.1186/s12906-015-0939-2)
Supplement: Additional file 3: Table 1. — Characteristics of 27 included studies on SM for chronic cor pulmonale heart failure. (DOC 136 kb) [file 12906_2015_939_MOESM3_ESM.doc]

**Table 1. Characteristics of 27 included studies on SM for chronic cor pulmonale heart failure**

| **Included studies** | **Sex**  **(M/F)** | **Sample**  **Size(T/C)** | **Averaged**  **Age(years)** | **Course of cor pulmonale** | **NYHA classification**  **from I to IV** | | | | | | **Treatment**  **group** | **Control group** | **Duration**  **(days)** | **Outcome Measures** | **ADEs**  **/ADRs** |
| --- | --- | --- | --- | --- | --- | --- | --- | --- | --- | --- | --- | --- | --- | --- | --- |
|  |  |  |  |  | **I** | | **II** | **III** | **IV** | |  |  |  |  |  |
| Zheng2014  [13] | 58/28 | 43/43 | 68. 8±8.6 | NA |  | | 28 | 38 | 20 | | SM+CON, 30ml  5%GS250ml,qd | CON | 14 | BNP  LVEF | NA |
| Jin2012  [14] | 76/44 | 60/60 | T:56.3±8.6  C:57.2±9.1 | T:22.5±1.8  C:21.5±2.0 |  | | T:28  C:26 | T:24  C:28 | T:8  C:6 | | SM+CON, 1.0ml/(kg·d)  2ml/min, qd | CON | 14 | LVEF BNP | NA |
| Zou2011  [15] | 30/24 | 27/27 | T:59.49±4.56  C:60.08±4.78 | 11. 0±1.1 |  | |  | T:16  C:15 | T:11  C:12 | | SM+CON, 100ml  qd | CON | 7-14 | NYHA | None |
| Xiao2009  [16] | 40/35 | 38/37 | T:70±8 C:70±8 | T:5-20  C:NA |  | |  | T:20  C:20 | T:18  C:17 | | SM+CON,30-45ml 5%GS250ml,qd | CON | 14 | NYHA  Death | T: A(3)  T: BC*(2) |
| Cheng2008  [17] | 66/8 | 37/37 | T:72.93±7.59 C:71.68±6.72 | T:16.78±8.14  C:15.93±7.84 |  | |  | T:9 C:NA | T:28 | | SM+CON, 40ml  5%GS/0.9%NS250ml  qd | CON | 14 | NYHA | None |
| Ye2008  [18] | 40/34 | 40/34 | T:70.5 C:71.3 | NA |  | |  | T:15  C:14 | T:25  C:20 | | SM+CON, 40ml  5%GS250ml,qd | CON | 7 | NYHA | T: D(1) |
| Guo2008  [19] | 70/45 | 64/51 | T:65±15 C:64±15 | NA | 115* | | | | | | SM+CON, 30ml  5%GS200ml,qd | CON | 10 | NYHA | None |
| Weng2008  [20] | 45/19 | 32/32 | T:69±7.2 C:70±7.8 | NA |  | | T:5  C:3 | T:20  C:21 | T:7  C:8 | | SM+CON, 60ml  5%GS250ml,qd | CON | 10 | NYHA | None |
| Wang2008  [21] | 57/23 | 40/40 | T:74±2 C:73±3 | T:12.5  C:13.3 |  | | T:9 C:8 | T:24  C:23 | T:7 C:9 | | SM+CON, 50ml  5%GS/0.9%NS250ml  qd | CON | 15 | NYHA  LVEF | None |
| Li2006  [22] | 42/22 | 32/32 | 62 | 8-30 | 64* | | | | | | SM+CON, 40ml  5%GS250ml,qd | CON | 10 | NYHA | NA |
| He2005  [23] | 31/13 | 23/21 | T:68 C:69 | T:8-12  C:8-11 |  | | T:3  C:2 | T:11 C:10 | T:9  C:9 | | SM+CON, 40ml  5%GS/0.9%NS250ml  qd | CON | 14 | NYHA  LVEF | None |
| Wu2003  [24] | 57/14 | 39/32 | T:64.9 C:64.3 | T:16.3  C:15.9 |  | | T:5  C:3 | T:18 C:16 | T:16  C:13 | | SM+CON, 30ml  5%GS250ml, qd | CON | 10 | NYHA | NA |
| Hu2003  [25] | 41/25 | 33/33 | T:63 C:62 | T:8-12  C:8-11 |  | | T:2  C:3 | T:15  C:15 | T:16  C:15 | | SM+CON, 40ml  5%GS250ml,qd | CON | 14 | NYHA | None |
| Shi2003  [26] | 43/35 | 42/36 | NA | NA |  | | 16 | 52 | 10 | | SM+CON, 10-60ml  5%GS250ml,qd | CON | 7-10 | NYHA | None |
| Jiang2003  [27] | 55/25 | 40/40 | T:69.80 C:69.41 | T:8-40  C:9-38 |  | | T:6  C:7 | T:24  C:24 | T:10  C:9 | | SM+CON, 40ml  5%GS250ml,qd | CON | 15 | NYHA | NA |
| Song1999  [28] | 66/8 | 37/37 | T:72.93±7.59 C:71.68±6.72 | T:16.78±8.14  C:15.93±7.84 |  | |  |  | T:37  C:37 | | SM+CON, 20ml  5%GS250ml,qd | CON | 7 | NYHA | NA |
| Xiao2011  [29] | 37/23 | 30/30 | T:56.5 C:57.3 | >10 |  | |  | T:17  C:16 | T:13  C:14 | | SM+CON, 30ml  5%GS250ml,qd | CON | 15 | NYHA PaO2  PaCO2 | T: E(1) |
| Guo1999  [30] | 43/15 | 36/22 | T:64.2 C:64.7 | NA |  | | T:4  C:3 | T:17  C:10 | T:15  C:9 | | SM+CON, 30ml  5%GS250ml, qd | CON | 10 | NYHA | NA |
| Peng1999  [31] | 41/19 | 30/30 | T:62 C:63 | NA |  | | T:5 C:5 | T:18  C:17 | T:7 C:8 | | SM+CON, 60-80ml  5%GS250ml,qd | CON | 14 | NYHA | None |
| Pang2012  [32] | 56/50 | 55/51 | T:57.6 C:56.3 | >2 |  | |  | T:28  C:25 | T:27 C:26 | | SM+CON, 30ml  5%GS/0.9%NS100ml  bid | CON | 15 | NYHA  PaO2  PaCO2 | T: E(1) |
| Chen2006  [33] | 32/26 | 30/28 | NA | NA |  | |  | T:16  C:15 | T:14  C:13 | | SM+CON, 50ml  5%GS200ml, qd | CON | 7 | NYHA | NA |
| Li2001  [34] | 80/37 | 57/60 | T:57.3 C:58.6 | NA |  | |  | T:40  C:42 | T:17  C:18 | | SM+CON, 30-40ml  qd | CON | 7-10 | NYHA | None |
| Chen2003  [35] | 52/26 | 40/38 | 73.5±8.7 | NA |  | | 15 | 59 | 4 | | SM+CON, 40ml  5%GS250ml,qd | CON | 14 | NYHA  LVEF | T: F(2) |
| Bao2001  [36] | 34/21 | 32/23 | T:57  C:60 | NA |  | |  | T:21  C:17 | T:11  C:6 | | SM+CON, 50ml  qd | CON | 10 | NYHA  PaO2  PaCO2 | NA |
| Xu2011  [37] | 55/35 | 46/44 | T:57.4 C:56.9 | NA |  | |  | T:21  C:18 | | T:25  C:26 | SM+CON, 60-100ml  5%GS/0.9%NS100-200ml  qd | CON | 10 | NYHA | T: C*(1)  C: GC*HF  (2) |
| Zhao2011  [38] | 39/41 | 40/40 | T:69.15±11 C:64.45±9.85 | T:1-8  C:1-10 | 6* | | | 38 | 36 | | SM+CON, 40ml  5%GS200ml, qd | CON | 14 | NYHA  PaO2  PaCO2 | None |
| Gu2001  [39] | 45/19 | 34/30 | T:58.43±17.32 C:57.96±17.14 | T:15.16±6.84  C:l5.38±7.02 | T:10  C:9 | T:18  C:17 | | T:6  C:4 |  | | SM+CON, 30ml  5%GS250ml,qd | CON | 14 | NYHA | NA |

Notes: SM: Shenmai injection; M: Males; F: Females; T: treatment group; C: control group; NA: not available; NYHA: New York Heart Association classification; LVEF: left ventricular ejection fraction; BNP: B-type natriuretic peptide; PaO2: Partial pressure of oxygen; PaCO2: Partial pressure of carbon dioxide; 115*: NYHA classification from II to IV; 64*: NYHA classification from III to IV; 6*: NYHA classification from I to II; NS: normal saline; GS: glucose; CON: conventional treatment; ADEs: adverse drug events; ADRs: adverse drug reactions; A: pain at the injection site; B: dizziness; C*: palpitation; D: dry mouth; E: rash; F: poor appetite; G: headache; H: nausea and vomiting.
